# Dronedarone-induced Phototoxicity in a Patient with Atrial Fibrillation

**DOI:** 10.7759/cureus.5731

**Published:** 2019-09-23

**Authors:** Praveen Datar, Paritosh Kafle, Frances M Schmidt, Bikash Bhattarai, Osama Mukhtar

**Affiliations:** 1 Internal Medicine, Interfaith Medical Center, Brooklyn, USA

**Keywords:** photosensitivity, dronaderone, rash

## Abstract

Dronedarone is a class III antiarrhythmic agent and a potent blocker of multiple intracardiac ion channels with many electrophysical properties common with amiodarone. Oral dronedarone, 400 mg twice daily, is approved for the maintenance of normal sinus rhythm in patients with a history of atrial fibrillation (AF) or atrial flutter. It is primarily used for the maintenance of sinus rhythm in patients with paroxysmal or persistent AF or atrial flutter. Dronedarone is a relatively new therapeutic agent which is a non-iodinated congener of amiodarone and hypothesized to have far lesser side effects. Photosensitivity is an uncommon side effect of dronedarone and not much has been described in the literature. Here we describe a patient with such complication.

## Introduction

Dronedarone is a class III antiarrhythmic agent and a potent blocker of multiple intracardiac ion channels with many electrophysical properties common with amiodarone [1].

Oral dronedarone, 400 mg twice daily, is approved for the maintenance of normal sinus rhythm in patients with a history of atrial fibrillation (AF) or atrial flutter. It is primarily used for the maintenance of sinus rhythm in patients with paroxysmal or persistent AF or atrial flutter and no evidence of heart failure or ventricular systolic function [2]. Dronedarone is a relatively new therapeutic agent, and there is still a paucity of data about its side effects. It is a non-iodinated congener of amiodarone, and because of the deletion of the iodine molecule, it is hypothesized that it will have far lesser side effects compared to amiodarone. As with any medication, there are potential side effects with dronedarone. These are mostly relatively benign and include crampy abdominal pain, diarrhea, nausea, and rash. Photosensitivity, while common in amiodarone, is not something which has been widely seen in patients taking dronedarone [3]. Here we describe a rare case of dermatologic complication of dronedarone.

## Case presentation

**Figure 1:**
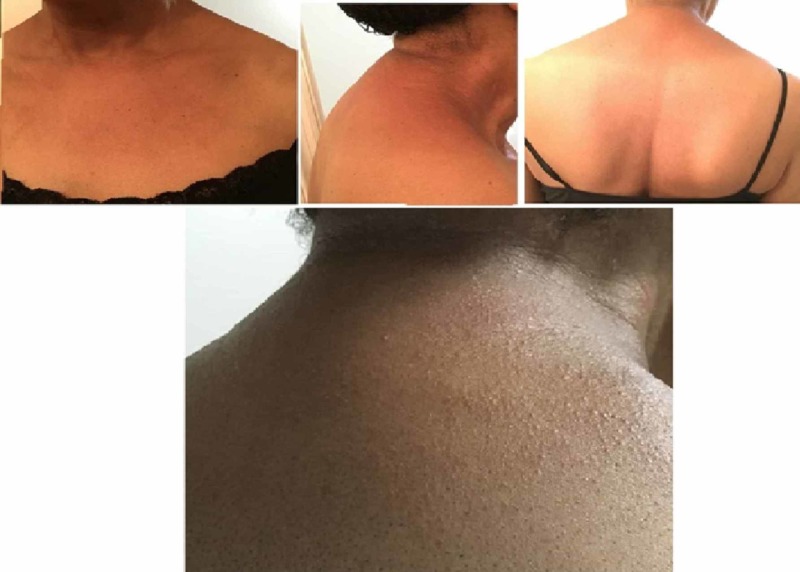
Erythematous maculopapular rash seen over the neck, bilateral forearms, and anterior chest

A 70-year-old African-American woman presented to the clinic complaining of pruritic reddish discoloration of her neck, chest, and both forearms after being exposed to the sun. Her medical history was significant for hypertension, AF, prior hospitalization for cerebrovascular accident (CVA) with no residual weakness, and with no known history of allergies. One month prior to presentation, she was admitted for CVA with left-sided hemiplegia. During her hospitalization, she was diagnosed with new-onset AF with a rapid ventricular response and was started on dronedarone for rhythm control along with Eliquis. She was later discharged home on aspirin, dronedarone, Eliquis, Lipitor, and losartan/hydrochlorothiazide (HCTZ). She mentioned that she had been exercising, walking for about three miles daily while being exposed to the sun. She stated that she otherwise felt well and had no additional complaints. Review of systems, likewise, was unremarkable. The physical examination revealed a cheerful elderly woman in no apparent distress. She was alert, awake, and orientated to time place and person. There appeared an in-confluent, well-demarcated, erythematous maculopapular rash over the neck, bilateral forearms, and anterior chest (Figure 1). The cardiovascular and respiratory examination was also normal. Routine laboratory investigations included complete blood count (CBC) and a comprehensive metabolic panel (CMP). Given the relation of the symptoms with dronedarone, it was discontinued. For the treatment of AF, she was started on metoprolol, and Eliquis was continued. She was advised to follow up in two weeks. Upon her follow-up, her CBC and CMP tests from two weeks back were normal, and her rash had entirely resolved.

## Discussion

Dronedarone is a benzofuran derivative related to amiodarone. Amiodarone use is limited by toxicity due to its high iodine content (pulmonary fibrosis, thyroid disease) as well as liver disease [2]. In dronedarone, the iodinated moieties are not present, reducing toxic effects on the thyroid and other organs. It is less lipophilic than amiodarone, and has a much smaller volume of distribution, and has an elimination half-life of 13-19 hours which is a stark contrast to amiodarone, which has a half-life of several weeks [2]. Upon reviewing five clinical trials conducted on the safety profile of dronedarone and evaluating 6,285 individuals, it was noted that adverse dermatologic reactions including eczema, allergic dermatitis, pruritus, and nonspecific rash occurred in 3% of placebo patients and 5% of dronedarone recipients with < 1% due to photosensitivity [4]. The lack of a definite biopsy in our case makes it very difficult to categorize the rash accurately; she was on HCTZ for hypertension which is a common cause of photosensitivity; however, she had been taking losartan/HCTZ combination for years without any side effects. However, the chronological association of the rash’s eruption following the initiation of dronedarone therapy and the disappearance of the rash following discontinuation of dronedarone makes it the likely cause. To our knowledge, this is the second reported case of dronedarone-induced phototoxicity in the literature.

## Conclusions

Limited data is available on the adverse drug effects of dronedarone as it is a relatively new drug approved for rhythm control of AF. While most of the side effects are benign, photosensitivity can be one of the concerning side effects of this medication.
